# The IGF1 small dog haplotype is derived from Middle Eastern grey wolves: a closer look at statistics, sampling, and the alleged Middle Eastern origin of small dogs

**DOI:** 10.1186/1741-7007-8-119

**Published:** 2010-09-08

**Authors:** Cornelya FC Klütsch, M Dominique Crapon de Caprona

**Affiliations:** 1 KTH-Royal Institute of Technology, Gene Technology, Stockholm, Sweden; 2 Department of Biology, University of Iowa, College of Liberal Arts and Sciences, Iowa City, IA, 52242, USA

## Abstract

This paper is a response to Gray MM, Sutter NB, Ostrander EA, Wayne RK: **The IGF1 small dog haplotype is derived from Middle Eastern grey wolves**. *BMC Biology *2010, **8**:16.

See research article at http://www.biomedcentral.com/1741-7007/8/16.

## Background

Linking the genetic basis of small body size in domestic dogs to closely related wolves may resolve the geographical origin of small-sized dogs. The recent *BMC Biology *article by Gray *et al. *[1] combines classical phylogeographical methods with single-nucleotide polymorphism (SNP) and sequencing data (6331 bp and 4811 bp) surrounding the insulin-like growth factor 1 (IGF1) gene on dog chromosome 15 to unravel the geographical origin of small dogs. The conclusion of the first data set including SNP data is mainly that 'grey wolves of Middle Eastern origin were slightly closer related to domestic dogs'. The central sequencing data set which is supposed to support the strong claim that the small dog haplotype (SDH) is derived from Middle Eastern wolves, consisted of a limited sample of 10 wolves (6331 bp sequence data set) and 28 wolves (4811 bp sequence data set), of which in the second data set, 8 were from Israel alone. We will discuss problems related to statistics, sample size and representativeness as well as highlight omissions in the discussion of the data. We thereby offer alternative explanations for the results presented by Gray *et al. *[1] and discuss this paper in the light of recent genetic findings in other studies. (Note that all figures, tables and additional files referred to throughout are those from the original paper.)

## Are the wolf sample sizes sufficient in the context of the study?

The sequencing data sets comprise two sets of different lengths (6331 bp/4811 bp) surrounding the region of the IGF1 gene. A central result highlighted in the article abstract based on the sequence data is that Middle Eastern grey wolf haplotypes show higher nucleotide diversity, and that these haplotypes therefore originated in this region (Table 2, [1]). There are several major problems with this interpretation of the data sets.

Firstly, a 'large sample of grey wolves' [1] for the DNA sequence data is in fact a scarce sample of 28 grey wolves (data set: 4811 bp; Table 2, [1]), of which 8 (28.6%) are from Israel. Only two samples from China, a region suggested as the origin of dog domestication [2] were sequenced. Obviously, this is not enough to evaluate if Chinese wolves have a higher or lower genetic diversity than Middle Eastern wolves and they could by pure chance be unrepresentative of their respective population (for example, closely related, immigrant from another population or an inbred/outcrossed specimen).

Secondly, regarding the point 'grey wolf samples were chosen to be globally distributed and representative of all major populations' [1], the authors omit to discuss that grey wolves were once distributed all over the Northern hemisphere [3] but are today distributed in patches throughout their former distribution range as the result of extermination, restricting the comparisons that can be made. It is therefore questionable whether a comparison to the current wolf population can give any tangible insight as to the diversity of wolf haplotypes at the time of domestication or, as the authors write, 'early in the history of domestic dogs'.

Thirdly, the largest remaining wolf populations of the Old World in North-East Asia (Kazakhstan, Russia, Siberia and China), which cover approximately 50% of Eurasia, are represented by only two samples in the sequence data set (Figure 1; four in the SNP data, [1]). However, this area should be well sampled in a phylogeographical study aiming at identifying the origin of the SDH.

Fourthly, Chinese wolves, like Middle Eastern wolves, are of small stature. In addition, they have a morphological feature ('"turned-back" apex of the coronoid process of the ascending ramus', [4]) typical of dogs. Chinese wolves must consequently be considered as a potential source for the SDH and should therefore be sampled in numbers equal to other wolf populations.

Finally, in a previous study (Additional file 2), Spanish wolves had the largest number (5) of haplotypes with greatest similarity to the SDH whereas Israeli wolves had only a single haplotype closely related to the SDH. However, only two Spanish wolves but eight Israeli wolves were chosen for sequencing. The reason for excluding the Spanish samples is not given in Gray *et al. *[1]. We therefore question how the samples were chosen for the analysis.

## Do Middle Eastern wolves show significantly higher nucleotide diversity?

The statement that Middle Eastern wolves show higher nucleotide diversity [1] is one of the main arguments used to conclude that Middle Eastern wolf haplotypes closely related to the SDH have originated in this region. This is based on nucleotide diversity combined with a standard deviation. The authors fail to provide the reader with any statistical test (*P *values) or confidence intervals for Middle Eastern nucleotide diversity to be significantly higher in comparison to all other regions. The standard deviations presented are large, partly exceed the estimated values (for example, Iran and China), and are consequently not sufficient to support the conclusion made. The central question is this: is the Israeli wolf population with a nucleotide diversity value (π) of 0.00055 (SD 0.00044; corrected for sample size) significantly different from populations of for example North America (π: 0.00043, SD: 0.00029), Europe (π: 0.00044, SD: 0.00029), Iran (π: 0.00166, SD: 0.00176), or China (π: 0.00010, SD: 0.00013)?

Furthermore, the authors did not test nucleotide diversity in the Spanish population separately from other European samples. This population harbours a high number of haplotypes (and five haplotypes similar to the SDH; Additional file 2 and p. 2) and therefore must be considered to be an alternative source of the SDH. The authors would have to rule out the Spanish wolf population as a source for the SDH in order to conclude that the Middle East is the source population. However, the results would be strongly affected anyway by inappropriate sampling sizes/sampling bias (see above) and thus may have resulted in significant results by pure chance.

Lastly, it is important to note that nucleotide diversity in wolf populations may not give any insight as to the origin of (small) dogs, because the nucleotide diversity among wolves may display other factors. For example, Middle Eastern wolves may have been a centre for diversification of wolf populations or Middle Eastern wolves may have had a larger effective population size than other populations. Both examples have no connection to (small-sized) dogs.

## Is the SDH derived from wolves in the Middle East?

The sequence data has also been used to reconstruct neighbour-joining trees (Figures 4 and 5, [1]) and minimum spanning networks (MSNs; Additional file 1, Figures S2 and S3, [1]). The SDH is indeed more closely related to Middle Eastern wolf haplotypes in the neighbour-joining trees/MSN. However, bootstrap values are generally low. More importantly, the five Spanish wolf haplotypes similar to the common SDH in a previous SNP study (Additional file 2) should have been sequenced and included to reconstruct neighbour-joining trees/MSN to test whether Spanish wolf haplotypes are an alternative origin for the SDH. However, only two Spanish samples were used (of which only one is actually closely related to the SDH) whereas eight Israeli samples (although only one was most similar to the SDH in Additional file 2) were studied.

The importance of comprehensive population/taxon sampling and character sampling for correct inference of evolutionary relationships is well known. However, the trees presented in Gray *et al. *[1] are based on rather few characters and, from looking at the principal component analysis (PCA) graph (Figure 3, [1]), one can see that character numbers (and thereby genetic variation) might have been considerably improved by sequencing longer DNA fragments; for example, the same region as represented by the dog-derived 94 SNP set used in the PCA. At the same time one may wonder how the trees/MSNs would have looked like with an improved sampling of populations. It is not known if these closest haplotypes can be found in other wolf populations as well. Just a few specimens for most wolf populations is simply not enough to exclude the possibility that other wolf populations carry a haplotype closely related to the SDH. The probability (*P*^−^) of losing a rare haplotype with frequency *q *in a sample of *n *individuals can be defined as *P*^− ^= (1 - q)^2n ^[5]. Therefore, even if the proportion of the closest haplotypes in a population is 40% to 20%, for example, there is a large possibility (13.0% to 41.0%) that no such haplotypes are included in two individuals representing the population, as in the case of Chinese (or Eurasian) wolves.

## The SNP data set

The last data set included SNP genotypes based on 94 dog-derived SNP markers spanning the IGF1 interval and resulted in a PCA (Figure 3, [1]) and a neighbour-joining tree (Additional file 1, Figure S1, [1]). From the PCA graph, the authors conclude a 'slightly closer kinship of Middle Eastern wolves to domestic dogs' (p. 2). A closer look at the PCA graph presented (Figure 3, [1]), reveals that the variation explained by the two axes is 31.5%; leaving 68.5% of the variation either unexplained or falling within groups. The clearest extractable result is that Akitas (as an ancient East Asian dog breed) are most closely related to wolves. The Akita included in this study exists in two sizes. The American Akita is derived from the ancient Japanese Akita, but has acquired a larger size, and is thus now considered to be another breed by the Japanese Kennel Club within the Fédération Cynologique Internationale. Since both breeds exist in North America, it would have been important to study the Akita samples according to the size of the dogs sampled. Because the Akita groups into two lots in Figure 3 [1], and only one of the groups is more closely associated with wolves, it would have been important to know if it is the smaller Japanese breed (clearly bred for hundreds of years outside the Middle East) that is closest to the Middle Eastern wolves but also to other Asian wolf samples. The most parsimonious conclusion here would have been that the Japanese Akita dog is related to Asian wolves and Middle Eastern wolves represent a fraction of Asian wolves.

Another point to be raised in this context is the usage of dog-derived SNPs and the potential effects on the results of this study. As pointed out in Morin *et al. *[6], downward biases in genetic diversity may arise when applying a SNP marker system in a supposedly high-diversity ancestral population, and SNP ascertainment bias has been proven to be potentially severe in wolves [7]. The dog-derived SNP panel used in Gray *et al. *[1] is, as the authors state, based on European dogs only. European dogs harbour only a small fraction of the total genetic diversity found in dogs, because breed formation included a bottleneck [8]. However, at the same time the most variable SNPs are selected from this gene pool. As a result, the SNP panel may be adequate for comparisons of genetic variation among European-bred dogs, but village dogs as well as the ancestor, the wolf, will harbour additional genetic variation not represented in the SNP panel. Consequently, comparisons between European dog breeds and village dogs or wolves will only capture variation and similarities relative to the European dog gene pool. Therefore, the wolf population appears as a small, compact cloud in the PCA analysis in Gray *et al. *[1]. However, this does not necessarily display the real relationships of wolf populations with each other or with domestic dogs.

## The influence of hybridisation on SNP analysis

Pang *et al. *[2] indicated that dog-wolf hybridisation happened in the Middle East and other studies [9,10] showed that Middle Eastern and Italian wolves hybridised with dogs and carry domestic dog DNA. It has to be pointed out that admixture of domestic dogs with Middle Eastern wolves would have happened after domestication [2], but before the diagnostic short interspersed nuclear element (SINE) and causal SNP mutation occurred in small dogs (otherwise wolves would carry the SINE and causal SNP). However, since no one knows when these mutations occurred, the potential influence of historical hybridisation for European and Middle Eastern wolf populations has to be considered. Therefore, it is equally likely that the Middle Eastern wolf population appears to be slightly more closely related to domestic dogs because of admixture instead of ancestry. In contrast, other wolf populations may appear only less related to domestic dogs, because they carry less/no domestic dog DNA or *vice versa *from hybridisations.

In fact, a more general problem in using autosomal genetic markers such as SNPs in the study of organisms that can hybridise with other species is the fact that even small influxes of DNA may result in a signal of similarity in subsequent analysis. It is simply unknown to date how much domestic dog DNA is carried by European and Middle Eastern wolves and since when, and how this 'dog DNA' has spread within the wolf or *vice versa*. However, mitochondrial DNA (mtDNA) [2] indicates that haplogroup d2 has spread into the Mediterranean and is present in today's European-bred dogs. Since the SNP panel has been selected from European dogs, it is also possible that some SNPs are actually originally Middle Eastern wolf SNPs, again offering an alternative explanation for the slightly closer relationship of domestic dogs and Middle Eastern wolves. The statistical analyses of SNP data might then be severely affected. It is striking that of all potential ancestral wolf populations, European (Spanish) and Middle Eastern wolves appear to show a slightly closer relationship to domestic dogs. In this context, the more general question must be raised regarding how much information can be extracted from a genetic comparison of domestic dogs with wolves? Finally, it should be noted that later introduced wolf DNA into the dog gene pool or *vice versa *will automatically also lead to increased nucleotide diversity estimates for dog-derived SNPs in this wolf population. The conclusion that an ancient origin of the small dog haplotype has to be in the Middle East is therefore in no way supported by the data.

Limiting the analysis to SNPs that amplify in both species and analysing SNP haplotypes may not necessarily cancel out severe bias effects. Combined with the considerably lower number of wolf samples in comparison to dog samples, variation and relationships among these species may be highly skewed in a PCA. Furthermore, only a few 'East Asian' (Chinese) wolves were included in this SNP data set and it seems that only a subset of the sequenced Middle Eastern wolves has been included. The relationship of (small) domestic dogs to Middle Eastern and East Asian wolves thus remains somewhat unclear.

Taken together, considering the issues regarding sample size, the choice of samples, the lack of significant nucleotide diversity levels, and the SNP bias, the presented data do not lend strong support to the conclusion of Gray *et al. *that the SDH is derived from Middle Eastern wolves in ancient times.

## Authors' contributions

Both authors contributed to the conception and coordination of this commentary. CFCK drafted the first text version and conducted literature research. MDCDC wrote and corrected the text. Both authors read, edited and approved of the final manuscript.
